# Engineered GO-Based Hydrogels for Controlled Hyaluronic Acid Release in Knee Osteoarthritis Treatment

**DOI:** 10.3390/polym18020152

**Published:** 2026-01-06

**Authors:** Roya Binaymotlagh, Damiano Petrilli, Laura Chronopoulou, Giorgio Mandato, Francesca Sciandra, Andrea Brancaccio, Marisa Colone, Annarita Stringaro, Leonardo Giaccari, Francesco Amato, Andrea Giacomo Marrani, Silvia Franco, Roberta Angelini, Cleofe Palocci

**Affiliations:** 1 Department of Chemistry, Sapienza University of Rome, Piazzale Aldo Moro 5, 00185 Rome, Italy; 2 Research Center for Applied Sciences to the Safeguard of Environment and Cultural Heritage (CIABC), Sapienza University of Rome, Piazzale Aldo Moro 5, 00185 Rome, Italy; 3 Institute of Chemical Sciences and Technologies “Giulio Natta”—SCITEC (CNR), Largo F. Vito, 00168 Rome, Italy; 4 School of Biochemistry, University of Bristol, Bristol BS8 1TD, UK; 5 National Center for Drug Research and Evaluation, Italian National Institute of Health, Viale Regina Elena, 299, 00161 Rome, Italy; 6 Institute for Complex Systems, National Research Council (ISC-CNR), Sapienza University of Rome, P.le A. Moro 2, 00185 Rome, Italy; 7 Physics Department, Sapienza University, P.le Aldo Moro 2, 00185 Rome, Italy

**Keywords:** peptide hydrogels, hyaluronic acid, graphene oxide, bioconjugate, osteoarthritis

## Abstract

Osteoarthritis (OA) is a prevalent chronic pain syndrome and a leading cause of disability worldwide, characterized by progressive deterioration of articular cartilage. This degradation leads to pain, swelling, inflammation, and eventual stiffness as the cartilage wears down, causing bone-on-bone friction. Current medical treatments primarily aim at pain relief; however, many interventions, especially invasive or surgical ones, carry risks of adverse outcomes. Consequently, intra-articular (IA) therapy, particularly hyaluronic acid (HA) injections, is widely adopted as a conservative treatment option. HA plays a crucial role in maintaining joint homeostasis by supporting proteoglycan synthesis and scaffolding, restoring optimal HA concentrations in synovial fluid, and providing chondroprotective and anti-inflammatory effects. In recent years, hydrogels composed of natural and synthetic materials have emerged as promising candidates for OA treatment. Our research focuses on the biosynthesis and characterization of novel hydrogel composites combining short peptide hydrogelators with aminated graphene oxide (a-GO) nanosheets functionalized with HA (a-GO-HA@Hgel). These a-GO-HA@Hgel nanocomposites are designed to facilitate the controlled release of HA into the extracellular matrix, aiming to promote cartilage regeneration and mitigate inflammation. The strategy is to exploit the oxygen-containing functional groups of GO nanosheets to enable covalent coupling or physical adsorption of HA molecules through various chemical approaches. The resulting a-GO-HA are incorporated within hydrogel matrices to achieve sustained and controlled HA release. We study the influence of a-GO-HA on the native hydrogel structure and its viscoelastic properties, which are critical for mimicking the mechanical environment of native cartilage tissue. Through this multidisciplinary approach combining advanced materials science and cellular biology, this work aims to develop innovative nanocomposite hydrogels capable of delivering HA in a controlled manner, enhancing cartilage repair and providing a potential therapeutic strategy for OA management.

## 1. Introduction

Osteoarthritis (OA), a major cause of disability in aging populations, is characterized by progressive degeneration of synovial joints—especially the knee, hip, and shoulder—leading to cartilage loss, subchondral bone remodeling, osteophyte formation, and chronic low-grade synovial inflammation. These changes result in persistent pain, stiffness, reduced mobility, and compromised quality of life [1,2]. OA pathophysiology involves mechanical overload, metabolic dysregulation, inflammatory signaling, and epigenetic mechanisms that disrupt chondrocyte homeostasis and extracellular matrix (ECM) stability [3]. Although current treatments, including nonsteroidal anti-inflammatory drugs (NSAIDs), corticosteroids, physiotherapy, and joint replacement, alleviate symptoms, they do not halt disease progression. Systemic therapies are limited by side effects and poor intra-articular bioavailability. Moreover, the avascular nature of cartilage hinders its intrinsic regenerative capacity [4].

Regenerative medicine offers strategies to restore native tissue architecture, with biomaterial-based scaffolds playing a central role in cartilage engineering by providing mechanical and biochemical cues that support cell adhesion, proliferation, and differentiation [5]. Natural biomaterials such as collagen, hyaluronic acid (HA), chitosan, fibrin, and alginate provide intrinsic biocompatibility, while synthetic polymers, including Poly(lactic-co-glycolic acid) (PLGA), Poly(ε-caprolactone) (PCL), and Polyethylene glycol (PEG), enable tunable mechanical properties and degradation rates [6,7].

HA is widely studied for its excellent biocompatibility, biodegradability, and multifunctionality as a viscosupplement and carrier of bioactive molecules [8]. Present in synovial fluid and cartilage ECM, HA supports lubrication, structural organization, and cell signaling via receptors such as Cluster of Differentiation 44 (CD44), a cell-surface glycoprotein receptor; Receptor for Hyaluronan-Mediated Motility (RHAMM); and Toll-Like Receptor 4 (TLR4), thereby influencing inflammation and repair. In OA, CD44 overexpression, together with reduced HA concentration and molecular weight, impairs synovial fluid viscoelasticity and worsens mechanical stress and inflammation [9,10]. Intra-articular HA injections are commonly used to restore lubrication and reduce pain [11]. HA’s molecular weight critically modulates residence time, rheological behavior, and biological activity: high molecular weight HA (>1500 kDa) provides prolonged joint retention and anti-inflammatory effects, whereas low molecular weight HA (<1000 kDa) enhances tissue penetration and endogenous HA synthesis but degrades faster. Very low molecular weight fragments (<300 kDa) may even exert pro-inflammatory effects [12,13]. Therefore, both high- and low-MW HA offer complementary therapeutic properties, and formulation choice should reflect disease severity and treatment goals [14].

Despite a favorable safety profile and effectiveness in early-to-mid OA, HA therapy is limited by rapid enzymatic and oxidative degradation within the joint, necessitating repeated injections [15]. These constraints underscore the need for HA delivery systems with enhanced stability and prolonged intra-articular retention [16]. Advanced platforms—including nanoparticles, hydrogels, liposomes, and HA-based matrices—enable sustained HA release and potential co-delivery of anti-inflammatory or disease-modifying agents, thereby improving therapeutic efficacy [17].

Nanocarrier design has become central to OA-targeted therapies. Graphene oxide (GO), a two-dimensional nanomaterial with a large surface area and abundant oxygen functional groups, offers versatile covalent and non-covalent binding for diverse therapeutics [18]. GO interacts with cellular and protein components, supporting applications in drug delivery, biosensing, imaging, and theranostics, particularly in oncology [19]. HA–GO conjugates combine the biocompatibility and receptor-targeting capacity of HA with the structural stability of GO, improving physiological stability and therapeutic potential. These nanocomposites have shown promise in anti-cancer applications and in managing meta-inflammation [20]. Their anti-inflammatory and targeting capabilities support potential use in OA for localized therapeutic delivery with reduced systemic effects [21].

Hydrogels—hydrophilic, water-rich polymer networks—are key platforms for controlled drug release because of their tunable porosity, biocompatibility, and ability to encapsulate diverse therapeutics [22,23]. Depending on the stabilizing interactions, hydrogels may be chemically or physically cross-linked, each offering distinct advantages in stability, injectability, and responsiveness [24,25]. Peptide-based hydrogels, formed by self-assembly of short sequences into supramolecular nanofibrous networks, provide reversible gelation, avoid toxic crosslinkers, and offer molecular-level customizability, supporting targeted, stimuli-responsive drug delivery and tissue integration [26,27,28]. However, their limited mechanical strength under physiological conditions challenges their use in load-bearing environments such as the joint [29]. Incorporating GO enhances hydrogel mechanical properties through π–π stacking, hydrogen bonding, and electrostatic interactions, creating additional physical crosslinking points that restrict polymer mobility, increase stiffness and toughness, and reduce excessive swelling [30,31]. These improvements support prolonged joint residence and mechanical resilience, making GO-based hydrogels promising candidates for OA therapy [32].

**Figure 1:**
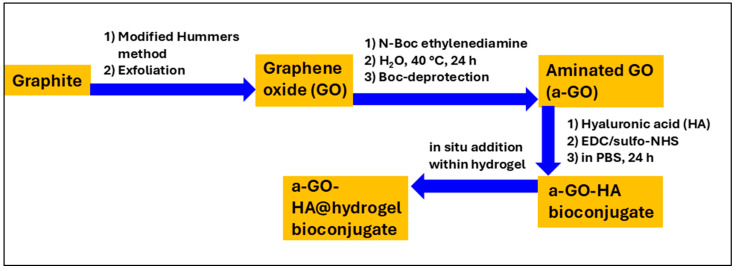
Schematic illustration of the preparation process of a-GO-HA@Hgel.

This study introduces an injectable peptide hydrogel integrated with aminated graphene oxide–hyaluronic acid (a-GO-HA) conjugates for potential intra-articular OA treatment. The hydrogel, formed via enzymatic biosynthesis of self-assembling tripeptides, incorporates a-GO-HA to enhance structural stability and therapeutic performance (Figure 1). a-GO offers amine- and oxygen-rich groups that form covalent and non-covalent interactions with HA, enhancing its stability and enzymatic resistance. These properties suggest strong potential for controlled, enzyme-mediated intra-articular release within the knee, which will be explored in future work [33,34]. Earlier studies indicate that GO-HA hydrogels maintain therapeutic HA levels for 5–8 days and reduce hyaluronidase-mediated degradation by up to 80%, outperforming free HA [35]. These features support their potential as localized, long-acting OA therapeutics with reduced dosing frequency and improved patient compliance. The composite hydrogels were characterized using X-ray Photoelectron Spectroscopy (XPS), Scanning Electron Microscopy (SEM) and Transmission Electron Microscopy (TEM) imaging. Their mechanical properties are evaluated using rheological measurements. In addition, their cyto-compatibility properties were assessed in vitro using SW1353 chondrosarcoma cells.

## 2. Materials and Methods

### 2.1. Materials

*N*-(9-Fluorenylmethoxycarbonyl)-phenylalanine (Fmoc-Phe, 99%, 387.44 g/mol) and diphenylalanine (Phe_2_, 98%, 312.36 g/mol) were purchased from Bachem GmbH (Weil am Rhein, Germany) and used as received. Hyaluronic acid, sodium salt, powder (MW ~1500 kDa, 98.4%), obtained from Contipro. The following compounds and all solvents were purchased from Sigma-Aldrich (St. Louis, MO, USA) and used as received: Lipase from *Pseudomonas fluorescens* (PFL ≥ 20,000 U/mg), graphite powder, sulfuric acid (37%, H_2_SO_4_), potassium permanganate (KMnO_4_), sodium nitrate (NaNO_3_), hydrogen peroxide (30%, H_2_O_2_), hydrochloric acid (12 M, HCl), N,N-dimethylformamide (DMF), N-BOC-ethylenediamine, methanol, *N*-(3-dimethylaminopropyl)-N′-ethylcarbodiimide hydrochloride (EDC), and *N*-hydroxysulfosuccinimide sodium salt (Sulfo-NHS).

### 2.2. Synthesis of Graphene Oxide (GO) and Functionalization with Amine Groups (a-GO)

Graphene oxide was synthesized by following an already reported procedure [36,37]. Briefly, 3.0 g of graphite powder was dispersed in 69 mL of H_2_SO_4_ under ice-bath stirring, after which 1.5 g of NaNO_3_ was added as an intercalating agent. Subsequently, 9 g of KMnO_4_ was slowly introduced as the oxidant. The reaction mixture was then diluted with 138 mL of water and maintained at 98 °C for 15 min. Oxidation was terminated by adding 420 mL of 30% H_2_O_2_. The product was vacuum-filtered, redispersed in ethanol, and centrifuged to remove residual water.

For amination, 100 mg of the prepared GO was dispersed in 100 mL of ultrapure water. The dispersion was sonicated for 30 min, and then 126 μL of N-Boc-ethylenediamine (en-Boc) was added. The mixture was stirred at 40 °C for 24 h. After the reaction, the suspension was centrifuged at 9000 rpm for 30 min. The resulting aminated GO (a-GO) was freeze-dried for 48 h. For deprotection of the amine group, 16 mL of hydrochloric acid (HCl) was diluted to a final volume of 50 mL with dioxane. The a-GO was dispersed in this solution and stirred at room temperature for 15 h. Following deprotection, the product was recovered by a series of centrifugations. The first centrifugation was conducted at 8000 rpm for 15 min, followed by six additional centrifugations in DMF (two times), methanol (two times), and ultrapure water (two times). Once the procedure was completed, the sample was freeze-dried for 48 h.

### 2.3. Preparation of a-GO-HA Conjugates

The conjugation process followed the previous report [38]. Briefly, 0.5 mg of a-GO was weighed and dispersed in 0.5 mL of deionized water in a vial. The dispersion was sonicated for about 15 min to ensure uniform distribution of a-GO. Meanwhile, a sodium salt of hyaluronic acid (HA) solution was prepared at 320 ng/mL. Then, 20 µL of EDC (5 mg/mL) and 40 µL of Sulfo-NHS (5 mg/mL) were added to the solution, and the mixture was stirred to activate the carboxylic acid groups on HA via Sulfo-NHS/EDC chemistry. After 30 min of activation, the HA solution was combined with the a-GO dispersion and allowed to react with continuous stirring at room temperature for 24 h. After the reaction, the mixture was divided into two 5 mL Eppendorf tubes and subjected to five centrifugation-washing cycles at 14,000 rpm for 30 min at 5 °C to remove unreacted components. The precipitate was then freeze-dried to remove residual water.

### 2.4. Preparation of a-GO-HA@Hgel Composite

40 µmol of Fmoc-phenylalanine and 40 µmol of diphenylalanine were dispersed in 1 mL of ultrapure distilled water in a glass vial and stirred with a magnetic stirrer. To prepare a peptide suspension, the pH was adjusted to 12 by adding 0.420 mL of 0.5 M NaOH, then stirred for about 10 min. The pH was then neutralized to ~7 by dropwise addition of 0.1 M HCl. The final volume was adjusted to 3 mL with distilled water. Next, 100 µL of *Pseudomonas fluorescens* lipase solution (50 mg/mL) was added to the peptide solution. The mixture was incubated in a thermostatic water bath at 37 °C for 30 min to initiate enzymatic hydrogelation. To create composite hydrogels, aqueous dispersions of a-GO or a-GO-HA conjugates were used instead of ultrapure distilled water.

### 2.5. Cell Culture and Viability Test

SW1353 human chondrosarcoma cells (ATCC #HTB-94) were cultured in DMEM supplemented with 10% fetal calf serum and antibiotics. When cells reached confluence, they were subcultured approximately twice weekly with 1% trypsin–EDTA at a 1:2 split ratio. 24-well tissue culture plates were coated with hydrogel composites and exposed to UV light in a laminar flow hood for 15 min, then conditioned overnight in DMEM. Cells were seeded at 2 × 10^4^ cells per well in DMEM. After 24 h, cell viability was assessed using the 3-(4,5-dimethylthiazol-2-yl)-2,5-diphenyltetrazolium bromide (MTT) assay, as described previously [39]. Briefly, cells were incubated with 0.5 mg/mL MTT reagent, which was converted into formazan crystals after about 3 h at 37 °C. Intracellular crystals were solubilized with 0.04 M HCl in isopropanol. The amount of formazan released into the culture supernatant was measured using an automatic microplate reader (BioTek 800 TS, Agilent, Santa Clara, CA, USA) at 562 nm. Each experiment was performed in triplicate and repeated three times; cell cytotoxicity was calculated using Equation (1), where OD represents the optical density.

(1) $$\%cell\;viability=\left(\frac{OD\;of\;the\;Sample}{OD\;of\;the\;Control}\right)\times 100\;$$

One-way analysis of variance (ANOVA) followed by Bonferroni’s multiple-comparison test was performed to determine the *p*-value using GraphPad Prism (8.0) software.

### 2.6. X-Ray Photoelectron Spectroscopy (XPS)

The XPS technique was used to investigate the chemical composition of GO and a-GO. To this end, 50 μL of aqueous dispersions of GO and a-GO were drop-cast onto freshly prepared H-terminated Si(100) wafers. XPS measurements were carried out using an Omicron NanoTechnology (Uppsala, Sweden) Multiprobe MXPS system equipped with a monochromatic Al Kα (hν = 1486.7 eV) X-ray source (Omicron XM-1000), operating the anode at 14 kV and 16 mA, and using a take-off angle of 21° with respect to the sample surface normal. The C 1s experimental spectra were curve-fitted to pseudo-Voigt functions with a 70:30 Gaussian–Lorentzian ratio, using a Shirley function for the secondary-electron background. The efficiency of the functionalization was determined by calculating the nitrogen-to-carbon ratio using Equation (2) [36]:

(2) $$N/C=\frac{A_{N\;1s}\times \;RSF_{C\;1s}\times \;\sqrt{KE_{C\;1s}}}{A_{C\;1s}\times \;RSF_{N\;1s}\times \sqrt{KE_{N\;1s}}}$$

where *Ai* is the spectral area of the region, *RSFi* is the relative sensitivity factor of the photoionized orbital (1 for C 1s and 1.77 for N 1s) [40], and *KEi* is the kinetic energy of the photoelectron (1202 eV for C 1s and 1086 eV for N1s).

### 2.7. Kaiser Test for the Detection of Free Primary Amine Groups on Solid Phases

The Kaiser colorimetric assay was used to detect free primary amine groups on solid-phase materials. Ninhydrin reacts with primary amines to produce a blue coloration, indicating unreacted amine sites and the extent of coupling reactions. The assay was performed using a commercial kit from Sigma-Aldrich and a Cary 50 UV-Vis spectrophotometer, following a procedure reported in the literature [41]. The absorbance of the resulting solution was measured at 570 nm using the UV-Vis spectrophotometer. The amount of free primary amine groups in the sample was quantified using Equation (3), which relates absorbance to micromoles of amine per gram of sample:

(3) $$\frac{µmol}{g}=\frac{Abs\;570\times Dillution\;factor\times V}{\varepsilon \times m}$$

where

- Abs 570 is the absorbance at 570 nm
- V is the final volume of the solution
- ε is the molar extinction coefficient
- m is the mass of the sample in grams

### 2.8. High-Performance Liquid Chromatography (HPLC)

The amount of HA conjugated to the a-GO surface was quantified using a Waters 1525 HPLC system with a dual λ detector (Waters 2487) and an OHpak SB-806M HQ column (8.0 mm I.D. × 300 mm L, Resonac Corporation, Tokyo, Japan). The flow rate was set to 1 mL/min at a pressure of 65 bar and a temperature of 40 °C; the mobile phase was water containing 0.1 M Na_2_SO_4_ [42]. The UV-Vis detector was set at 198 nm, and an approximate retention time of 6.7 min was obtained for HA.

### 2.9. Electron Microscopy

Field-emission scanning electron microscopy (FE-SEM) analyses were performed at 5 kV. Samples were deposited onto 13 mm glass coverslips and air-dried. They were then chromium-coated by sputtering (TurboQ High-Vacuum Coating System, Quorum Laughton, Lewes, UK) and observed using an FEI Quanta Inspect FEG scanning electron microscope (FEI, Waltham, MA, USA) [43]. Transmission electron microscopy (TEM) observations were carried out using a Philips EM208S (FEI) equipped with a tungsten filament electron source and a maximum magnification of ×200,000. Images were acquired with a MegaView III system (Olympus Soft Imaging Solutions, Münster, Germany). TEM specimens were prepared by depositing a drop of the a-GO or HA-functionalized a-GO suspension onto a 400-mesh copper grid coated with a formvar/carbon film, negatively stained with 1% phosphotungstic acid, and subsequently examined under the TEM [44].

### 2.10. Rheology Measurement

Rheological characterization of the hydrogel samples was performed using a rotational rheometer (MCR 102, Anton Paar, Graz, Austria) with a cone–plate geometry (plate diameter of 24.964 mm, cone angle of 1.998°, and truncation gap of 104 μm). Oscillatory measurements were performed in frequency sweep mode to determine the storage modulus (G′) and loss modulus (G″) as a function of angular frequency at a fixed strain amplitude of 0.5% within the linear viscoelastic regime. Temperature was controlled with a Peltier system, and solvent evaporation was minimized using an evaporation blocker and an isolation hood. All measurements were conducted at a controlled temperature of 25 °C [45].

### 2.11. Swelling Ability Studies

The swelling behavior of the hydrogels was assessed according to our previous report [46]. Briefly, each sample was immersed in 3 mL of phosphate-buffered saline (PBS, pH 7.4) and incubated at 37 °C for 24 h. After incubation, excess PBS was carefully removed, and the swollen weight of each hydrogel (Ws) was recorded. The samples were then freeze-dried, and their dry weights (Wd) were measured. The swelling ratio (q) was calculated using Equation (4):

(4) $$q=\frac{\left(Ws-Wd\right)}{Wd}$$

## 3. Results and Discussion

### 3.1. Graphene Oxide Functionalization Approach

**Figure 2:**
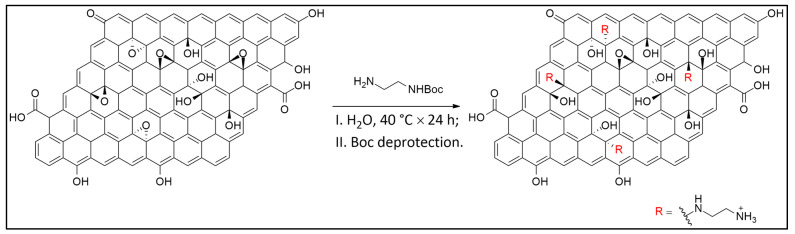
Reaction route to a-GO.

The experimental methodology in this study was organized into four main stages: (i) synthesis of GO from commercial graphite, (ii) chemical amination of the GO surface, (iii) conjugation of the aminated GO with the sodium salt of hyaluronic acid (HA), and (iv) insertion of the resulting nanostructures into peptide-based hydrogels to create a targeted nanocomposite system (see Figure 1). Amination of GO is a key step that enables further bioconjugation with functional biomolecules such as HA. In this work, the amination process added primary amine (–NH_2_) groups to the GO surface, yielding an aminated GO (a-GO) derivative (Figure 2). The N-Boc protecting group is crucial to prevent unwanted side reactions, such as self-condensation or crosslinking. After the initial amination reaction, a deprotection step was performed under acidic conditions to remove the Boc group and reveal the free primary amines. Given the known reactivity of amines, reaction of the free aminic end-group of en-BOC with epoxide groups of pristine GO is expected, possibly leading to basal covalent C–N bonds after ring-opening.

The presence and quantity of primary aliphatic amine groups introduced onto the GO surface were first evaluated using the colorimetric Kaiser test, a well-established method for detecting free primary amines that forms a blue chromophore (Ruhemann’s purple) with ninhydrin under alkaline conditions. The assay revealed an amine content of 150 ± 5 µmol/g, indicating a significant degree of functionalization (Figure S1). This result provides quantitative evidence for the successful covalent attachment of ethylenediamine (en) moieties to the GO framework. To determine the chemical composition of the pristine GO and a-GO samples, XPS spectra were acquired in the C 1s and N 1s photoionization regions.

**Figure 3:**
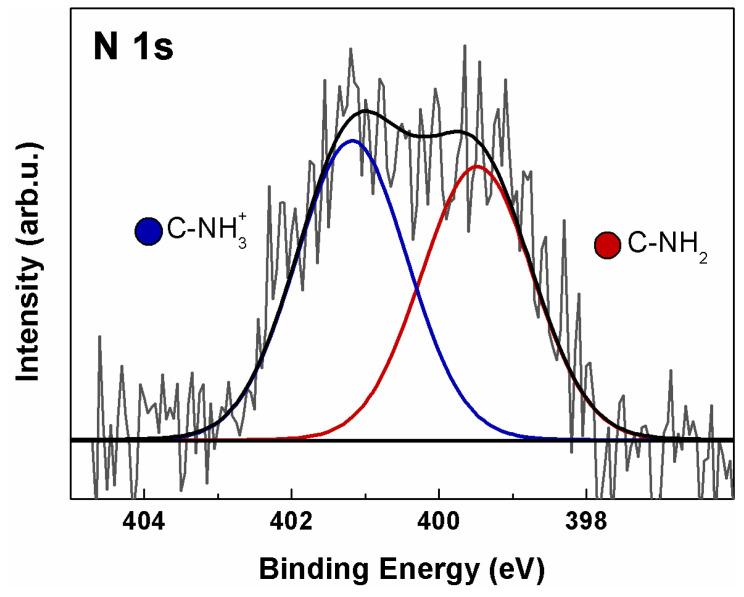
XPS N 1s spectrum of a-GO. Continuous colored curves are the results of curve-fitting reconstruction.

Analysis of the N 1s ionization region of a-GO (Figure 3) revealed the presence of N associated with two distinct chemical environments, assigned to the products of the reaction between GO and en. In fact, a contribution is observed at 399.5 eV (green), associated with terminal amines (C–NH_2_) and secondary aminic N atoms directly bonded to the basal GO C atoms (C–NH–C) [47,48]. A possible contribution from edge C=N (imine) groups cannot be excluded, though it is difficult to ascertain because the N 1s chemical shift is very close to that of aliphatic amines [40]. A further equally intense component is also present at 401.2 eV (blue), associated with protonated terminal aminic groups (C–NH_3_^+^), probably due to residual HCl from the deprotection step [49]. The N/C ratio has been calculated via Equation (2), yielding a value of 0.022, corresponding to an atomic N% of 2.2 with respect to total carbon. Considering that en has two N atoms, a 1.1% functionalization is obtained.

**Figure 4:**
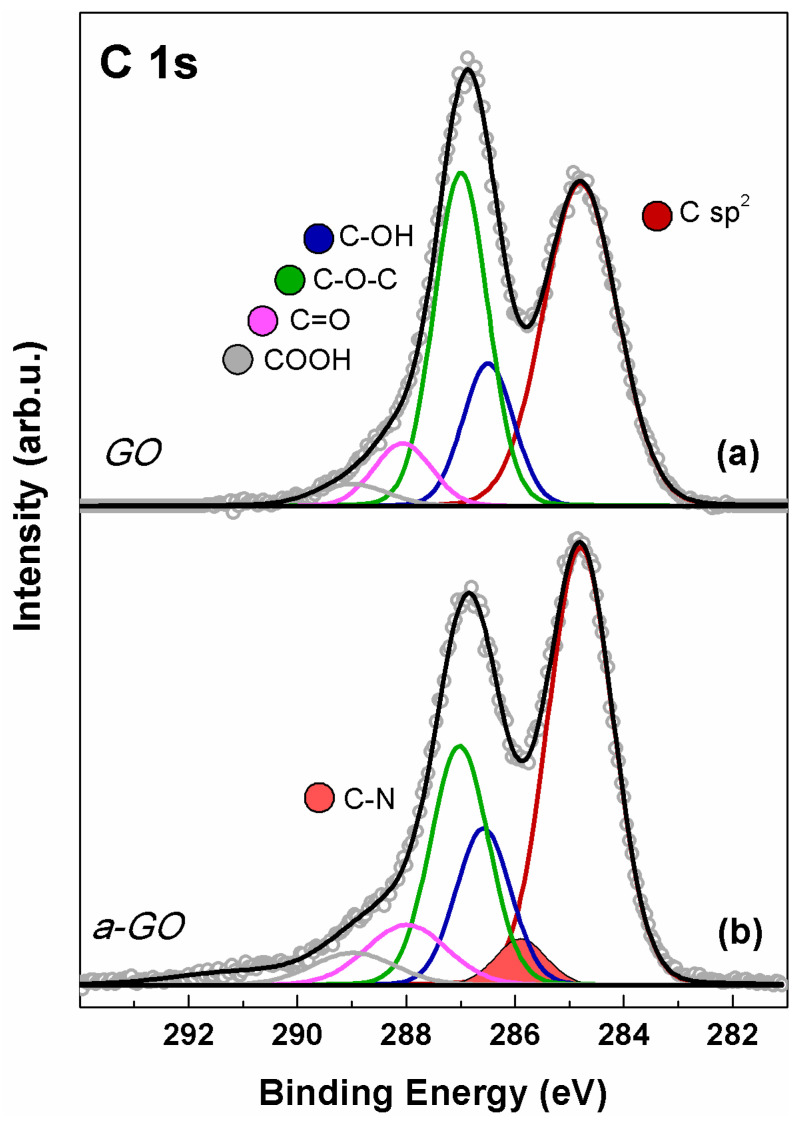
XPS C 1s spectrum of (a) GO and (b) a-GO. Continuous colored curves are the results of curve-fitting reconstruction.

The C 1s spectra of GO and a-GO are shown in Figure 4. In both spectra, five distinct components fit the experimental data, peaking at 284.8, 286.5, 287.0, 288.1, and 288.9 eV BE. These peaks are assigned to C=C and C=C–H sp^2^-hybridized carbon (red), hydroxyl (blue), epoxide (green), carbonyl (magenta), and carboxyl (gray) functional groups [34,50]. The C 1s spectrum of a-GO (Figure 4b) was fitted using an additional contribution at 285.8 eV (orange-filled), consistent with C–N bonds in both aliphatic aminic and imine groups [51]. In a-GO, a slight decrease in intensity in the 286–287 eV range is observed compared to GO, due to a diminished epoxide contribution, partially offset by increases in C–OH and C–N signals. This is consistent with the expected results of epoxide ring-opening upon reaction with en-BOC.

Overall, XPS results support the presence of free amine groups, indicating successful surface functionalization of GO. This functionalized a-GO material serves as an intermediate for subsequent conjugation with HA.

### 3.2. Characterization of a-GO-HA Conjugate

To quantify the amount of HA conjugated to the a-GO nanosheets, the concentration of unbound HA remaining in the reaction medium after 24 h of incubation was determined by HPLC. Briefly, the supernatant containing residual free HA was injected into the HPLC system, and the area of the UV absorbance peak corresponding to HA was recorded. Using a calibration curve (Figure S2), the concentration of free HA was determined, and the amount of HA conjugated to the a-GO nanosheets was calculated. Conjugation efficiency was estimated to be approximately 20% (for 0.50 mg/mL of a-GO, 0.10 mg/mL absorbed HA), with smaller peak areas corresponding to higher HA attachment onto the nanosheet surface.

**Figure 5:**
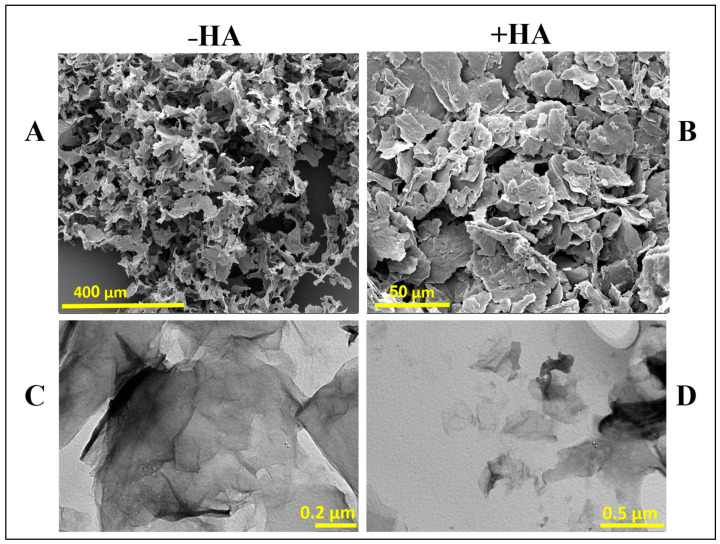
SEM (**A**,**B**) and TEM (**C**,**D**) images of aminated graphene oxide (a-GO) before and after HA conjugation. –HA and +HA represent the absence and presence of HA, respectively.

Figure 5 shows SEM and TEM images of aminated graphene oxide (a-GO) nanosheets before and after conjugation with HA, respectively. As shown in the SEM images (Figure 5A,B), bare a-GO exhibits a characteristic ultrathin, layered morphology with a dry, flaky texture (Figure 5A). After HA conjugation (Figure 5B), noticeable morphological changes are observed, including a moderate increase in sheet thickness and a less-defined surface texture. The edges of the nanosheets appear more diffuse and blurred, indicating successful surface modification. These morphological alterations likely result from the adsorption and/or covalent attachment of HA chains onto the a-GO surface, leading to partial coverage of the nanosheets and masking of sharp structural features. Such changes are consistent with the formation of a hydrated polymer layer on the GO surface, which contributes to the observed increase in opacity and edge fuzziness under SEM imaging. In the TEM images (Figure 5C,D), the aminated graphene oxide (a-GO) nanosheets show their typical thin, wrinkled, sheet-like appearance with clearly defined edges (Figure 5C). The high transparency of the sheets highlights their ultrathin structure. After conjugation with HA (Figure 5D), the nanosheets become slightly darker and less transparent, suggesting that HA molecules have been successfully attached to the graphene surface. Size changes from 63 nm to 384 nm may reflect this HA coating. The edges also appear smoother and somewhat blurred compared to the bare a-GO, which points to the presence of a coating layer [52]. Overall, the TEM observations, together with the SEM results, confirm that HA functionalization effectively modifies the surface of a-GO, leading to subtle but noticeable morphological changes.

The morphological changes observed in SEM and TEM, including thicker nanosheets, reduced transparency, and slightly blurred edges, align with previous reports on HA-coated GO systems and further confirm the successful conjugation of HA to the graphene surface [52].

### 3.3. Characterization of the a-GO-HA@Hgel Nanocomposite

**Figure 6:**
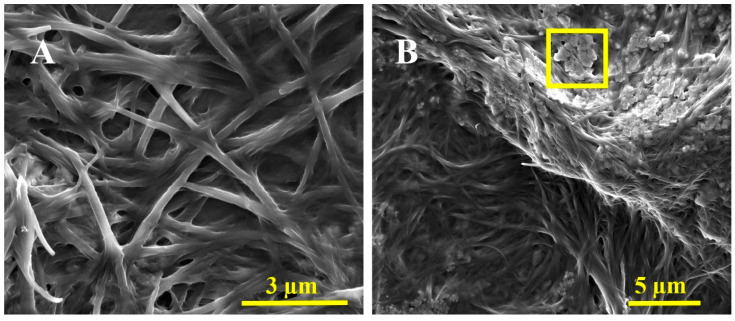
Representative SEM images of the (**A**) pristine Hgel and (**B**) a-GO-HA@Hgel conjugate. The yellow square indicates a typical a-GO-HA lamella.

We have submitted our a-GO-HA@Hgel nanocomposite to various assays to assess its structural integrity, surface morphology, and successful component integration. SEM (Figure 6) showed that the peptide hydrogel’s characteristic fibrillar network was preserved in both the original and nanocomposite samples. However, SEM images revealed structural differences between the pristine peptide hydrogel (Figure 6A) and the a-GO-HA@Hgel nanocomposite (Figure 6B). Both samples maintained the fibrillary network typical of peptide hydrogels, but the nanocomposite exhibited a denser, more interconnected structure. This increased density likely results from the successful incorporation of aminated-GO-hyaluronic acid nanoparticles into the hydrogel matrix. In the nanocomposite, the nanoparticles appeared embedded within or attached to the fibrillary structure, indicating physical interactions between the peptide network and the a-GO-HA conjugates. These morphological changes suggest that, although the native hydrogel fibrillary structure remains intact, the presence of nanoparticles enhances the overall complexity of the material and could ultimately improve the mechanical and functional properties of the hydrogel system.

The fibrillar structure of the hydrogel is well preserved after incorporating a-GO-HA, consistent with previous reports on graphene-reinforced peptide and polymeric hydrogels. In these systems, adding graphene-based nanofillers strengthens the network by improving connectivity without disrupting the hydrogel’s self-assembly. The denser, more interconnected morphology observed in the present system closely resembles other GO-based nanocomposite hydrogels, where graphene sheets act as physical crosslinkers, fostering additional interactions between fibrils and ultimately enhancing the overall structural cohesion of the network [53].

**Figure 7:**
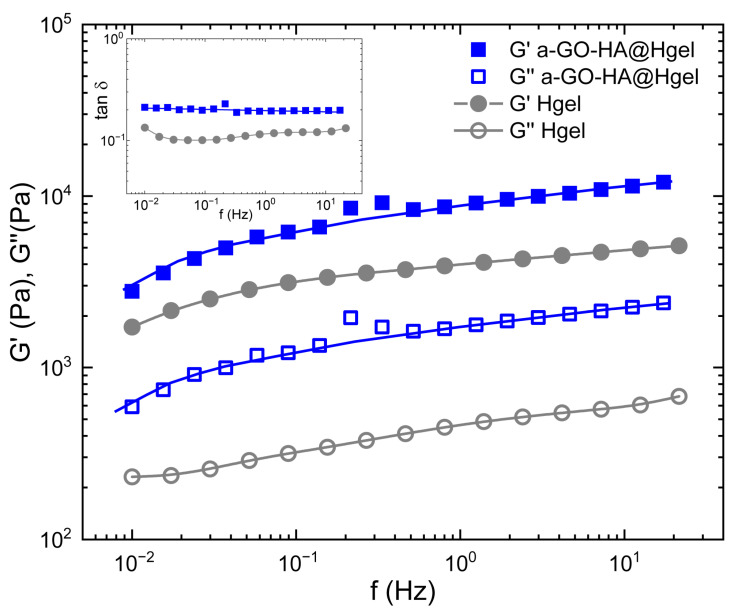
Frequency sweep and loss factor (inset) measurements of pristine Hgel and a-GO-HA@Hgel. Lines are guides for the bare eye.

Rheological measurements were performed to evaluate the viscoelastic properties and mechanical strength of both the pristine peptide hydrogel and the composite hydrogel containing a-GO-HA (Figure 7). Frequency sweep tests were conducted within the linear viscoelastic region, where small oscillatory deformations enable accurate probing of the material’s internal structure. The storage modulus (G′), which indicates elastic or solid-like behavior, and the loss modulus (G″), which indicates viscous or liquid-like behavior, were measured. Both hydrogels exhibited typical gel-like behavior, with G′ consistently higher than G″ throughout the entire frequency range [54]. Moreover, the loss factor (tanδ = G″/G′), reported in the inset of Figure 7, remained well below 1 across the entire frequency range, further confirming the predominantly elastic nature of these materials. A loss factor below unity indicates that energy storage dominates over energy dissipation during deformation, a hallmark of stable, solid-like hydrogel networks. This behavior suggests that both systems possess a well-percolated structure capable of sustaining shape and resisting flow even under dynamic mechanical perturbations. This evidence demonstrates that the materials behave more like soft solids rather than flowing liquids, a characteristic of hydrogel systems with a well-developed internal network structure [54]. The addition of a-GO-HA to the peptide hydrogel clearly increased both G′ and G″ values. The higher storage modulus suggests a stiffer network with a greater ability to store mechanical energy, while the increase in loss modulus indicates more energy dissipation under stress. Therefore, the composite hydrogel exhibited a higher loss factor than the pristine one. This indicates that, alongside enhanced stiffness, the composite also displays a stronger viscous contribution and more effective energy dissipation mechanisms. This combined rise in both elastic and viscous components shows a significant improvement in mechanical toughness compared to the pristine hydrogel. This enhancement can be attributed to the synergistic effects of the a-GO-HA within the network. The a-GO provides additional non-covalent interactions, such as hydrogen bonding and electrostatic attractions with the peptide chains, which help strengthen the network structure. Meanwhile, the HA component enhances steric entanglements and hydration-mediated interactions, further stabilizing the matrix. Together, these effects create a more robust and resilient hydrogel that can better distribute mechanical loads and resist deformation under shear.

The swelling behavior of hydrogels is a critical parameter that reflects their network structure, crosslinking density, and interactions with water molecules. As shown in Table 1, the swelling degree of the a-GO-HA@Hgel was found to be significantly lower than that of the pristine hydrogel, indicating a denser and more constrained polymeric network. This reduction in swelling can be attributed to the incorporation of a-GO, which likely introduces additional physical or chemical crosslinking points within the hydrogel matrix. The presence of a-GO enhances interfacial interactions between the graphene oxide sheets and the polymer chains, thereby limiting the hydrogel’s ability to absorb water excessively. Interestingly, this behavior is in inverse correlation with the rheological properties of the hydrogels, as shown in Figure 7. While the swelling degree decreased with a-GO incorporation, the mechanical strength—as reflected by rheological measurements—was enhanced. This inverse relationship suggests that the tighter network formed in the a-GO-HA@Hgel restricts swelling while simultaneously improving structural integrity and resistance to deformation. These findings support the role of a-GO not only as a reinforcing agent but also as a modulator of the hydrogel’s water uptake behavior, providing a balance between mechanical performance and swelling capacity suitable for various biomedical or soft material applications.

The lower swelling degree observed for the a-GO-HA@Hgel compared to the pristine hydrogel is in good agreement with previous studies on graphene oxide–based hydrogel systems. In these systems, the incorporation of GO nanosheets is known to increase the effective crosslinking density of the network, thereby limiting polymer chain mobility and reducing water uptake. The inverse relationship between swelling behavior and mechanical reinforcement observed in this work is a well-established characteristic of nanocomposite hydrogels, underscoring the dual role of a-GO-HA as both a reinforcing component and a modulator of hydrogel hydration [55].

### 3.4. Cell Viability Assay

**Figure 8:**
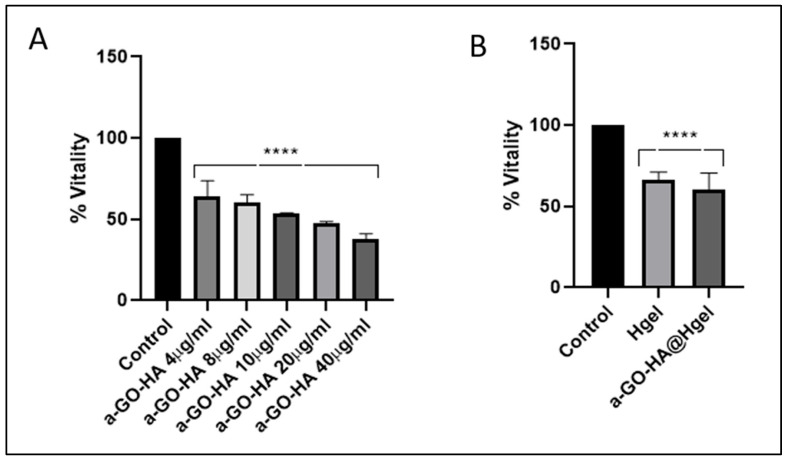
Vitality of SW1353 cells cultured on (**A**) different concentrations of a-GO-HA NPs and (**B**) a-GO-HA@Hgel composites. Data are expressed as percentage of cell vitality relative to control cells grown on uncoated wells. **** represents statistically significant differences with control (*p* < 0.0001).

The biocompatibility of the a-GO-HA NPs and their corresponding hydrogel composites was evaluated using SW1353 chondrosarcoma cell line, a widely accepted in vitro model for osteoarthritis-related studies due to its phenotypic resemblance to human chondrocytes under specific culture conditions [56]. Cells were coated on a-GO-HA NPs and a-GO-HA@Hgel composites, and viability was measured by standard metabolic assay after 24 h of cell growth. a-GO-HA NPs were tested at different concentrations (from 4 µg/mL to 40 µg/mL) and showed a significant, concentration-dependent reduction in cell vitality (Figure 8A). Therefore, we selected 8 µg/mL, corresponding to a ~40% decrease in cell viability, which would represent a good compromise to ensure detectable biological effects without excessive cytotoxicity. Further viability assays confirmed the cytocompatibility of 8 µg/mL a-GO@Hgel composite, in agreement with previous findings [24], and confirmed that a-GO-HA@Hgel did not drastically reduce cell vitality (Figure 8B). The viability of SW1353 cells cultured on a-GO-HA@Hgel was maintained at approximately 70% relative to control cells grown on uncoated wells, a level generally regarded as indicative of acceptable biocompatibility.

## 4. Conclusions

In this study, we developed and characterized the a-GO-HA@Hgel nanocomposite as a platform for controlled hyaluronic acid (HA) delivery in knee osteoarthritis. XPS confirmed the successful formation of aminated graphene oxide (a-GO), and HPLC showed that HA was conjugated with an efficiency of about 20%. SEM and TEM analyses clearly demonstrated the impact of HA functionalization: the nanosheets became slightly thicker, less transparent, and showed smoother, blurred edges, consistent with the presence of a hydrated HA coating. The hydrogel structure was also influenced by nanoparticle incorporation, with SEM revealing a denser and more interconnected fibrillar network while still preserving the native hydrogel architecture. Mechanical and swelling studies further supported the material’s suitability by showing improved stability, viscoelasticity, and water retention, all important features for sustained HA release. Biological tests indicated that, although free a-GO reduced cell viability in a dose-dependent manner, embedding the nanoparticles in the hydrogel greatly mitigated this effect, maintaining roughly 70% viability in SW1353 cells. Taken together, these findings show that a-GO-HA@Hgel composites combine structural integrity, controlled HA delivery, and acceptable cytocompatibility, making them a promising candidate for minimally invasive osteoarthritis therapy.

Complementary ex vivo studies using synovial fluid or cartilage explants, along with mathematical or computational models of diffusion and degradation, could help refine key experimental parameters before progressing to animal studies. Future work should also aim at optimizing the amount of HA conjugated to the nanoparticles to better adapt the system for in vivo application. Ultimately, establishing the in vivo release profile of the nanocomposite will be crucial for moving this platform toward clinically relevant osteoarthritis treatments.

## Figures and Tables

**Table 1:** Swelling degree (q) of the pristine Hgel and a-GO-HA@Hgel.

Type of Hydrogel	q
Pristine Hgel	78.90 ± 0.22
a-GO-HA@Hgel	57.05 ± 0.05

## Data Availability

All data generated or analyzed during this study are included in this published article and its Supplementary Materials.

## Supplementary Materials

The following supporting information can be downloaded at: https://www.mdpi.com/article/10.3390/polym18020152/s1, Figure S1: UV-vis spectrum resulting from the Kaiser test on a-GO; Figure S2: HPLC calibration curve of HA (5–100 ppm).
