# Supplementary material for: Engineered GO-Based Hydrogels for Controlled Hyaluronic Acid Release in Knee Osteoarthritis Treatment
Source: Polymers (Basel). 2026 Jan 6;18(2):152. doi: 10.3390/polym18020152 (PMC12845103; doi:10.3390/polym18020152)
Supplement: Supplementary file 1 [file polymers-18-00152-s001.zip › polymers-4063088-supplementary.pdf]

Supporting Information (SI) of

Engineered GO-Based Hydrogels for Controlled Hyaluronic Acid Release in Knee Osteoarthritis Treatment

Roya Binaymotlagh [1], Damiano Petrilli [1], Laura Chronopoulou [1,2], Giorgio Mandato [1], Francesca Sciandra[3], Andrea Brancaccio [3,4], Marisa Colone [5], Annarita Stringaro [5], Leonardo Giaccari [1], Francesco Amato [1], Andrea Giacomo Marrani [1], Silvia Franco [6,7], Roberta Angelini [6,7] and Cleofe Palocci [1,2,*]

1 Department of Chemistry, Sapienza University of Rome, Piazzale Aldo Moro 5, 00185 Rome, Italy;
2 Research Center for Applied Sciences to the Safeguard of Environment and Cultural Heritage (CIABC), Sapienza University of Rome, Piazzale Aldo Moro 5, 00185 Rome, Italy
3 Institute of Chemical Sciences and Technologies "Giulio Natta"—SCITEC (CNR), Largo F. Vito, 00168 Rome, Italy;
4 School of Biochemistry, University of Bristol, Bristol BS8 1TD, UK
5 National Center for Drug Research and Evaluation, Italian National Institute of Health, Viale Regina Elena, 299, 00161 Rome, Italy;
6 Institute for Complex Systems, National Research Council (ISC-CNR), Sapienza University of Rome, P.le A. Moro 2, 00185 Rome, Italy;
7 Physics Department, Sapienza University, P.le Aldo Moro 2, 00185 Rome, Italy
* Correspondence: cleofe.palocci@uniroma1.it

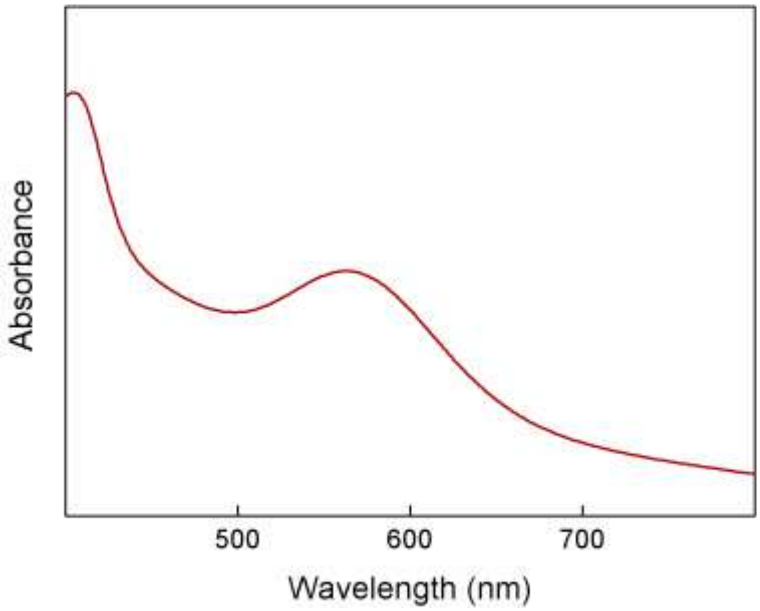

Figure S1. UV-vis spectrum resulting from the Kaiser test on a-GO.

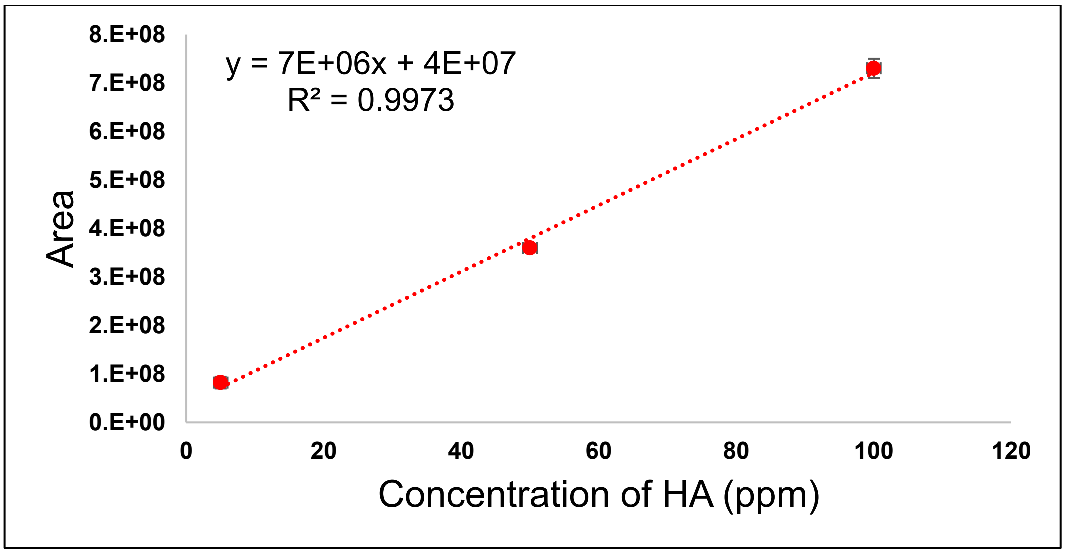

Figure S2. HPLC calibration curve of HA (5-100 ppm).
